# Automated Identification of River Hydromorphological Features Using UAV High Resolution Aerial Imagery

**DOI:** 10.3390/s151127969

**Published:** 2015-11-04

**Authors:** Monica Rivas Casado, Rocio Ballesteros Gonzalez, Thomas Kriechbaumer, Amanda Veal

**Affiliations:** 1 School of Energy, Environment and Agrifood, Cranfield University, Cranfield MK430AL, UK; E-Mail: t.kriechbaumer@cranfield.ac.uk; 2 Regional Centre of Water Research Centre (UCLM), Ctra. de las Peñas km 3.2, Albacete 02071, Spain; E-Mail: rocio.ballesteros@uclm.es; 3 Hydromorphological Team, Environment Agency, Manley House, Kestrel Way, Exeter, Devon EX27LQ, UK; E-Mail: amanda.veal@environment-agency.gov.uk

**Keywords:** Unmanned Aerial Vehicle, photogrammetry, Artificial Neural Network, feature recognition, hydromorphology

## Abstract

European legislation is driving the development of methods for river ecosystem protection in light of concerns over water quality and ecology. Key to their success is the accurate and rapid characterisation of physical features (*i.e.*, hydromorphology) along the river. Image pattern recognition techniques have been successfully used for this purpose. The reliability of the methodology depends on both the quality of the aerial imagery and the pattern recognition technique used. Recent studies have proved the potential of Unmanned Aerial Vehicles (UAVs) to increase the quality of the imagery by capturing high resolution photography. Similarly, Artificial Neural Networks (ANN) have been shown to be a high precision tool for automated recognition of environmental patterns. This paper presents a UAV based framework for the identification of hydromorphological features from high resolution RGB aerial imagery using a novel classification technique based on ANNs. The framework is developed for a 1.4 km river reach along the river Dee in Wales, United Kingdom. For this purpose, a Falcon 8 octocopter was used to gather 2.5 cm resolution imagery. The results show that the accuracy of the framework is above 81%, performing particularly well at recognising vegetation. These results leverage the use of UAVs for environmental policy implementation and demonstrate the potential of ANNs and RGB imagery for high precision river monitoring and river management.

## 1. Introduction

Environmental legislation [1,2,3] aiming to improve the quality of riverine ecosystems has driven the development of a vast number of methods for the hydromorphological assessment of rivers [4]. Within this context, hydromorphology refers to the physical characteristics of the shape, boundaries and content of a river [1]. There are currently over 139 different hydromorphological assessment methods used to characterise both physical in-stream and riparian habitat, river channel morphology, hydrological regime alteration or longitudinal river continuity. In Europe alone, the implementation of the Water Framework Directive (WFD [1]) has led to the development and use of over 73 methodologies [4] such as the LAWA method in Germany [5] or the CARAVAGGIO assessment in Italy [6]. In the United Kingdom (UK), the key methods adopted are the River Habitat Survey (RHS) [7,8] and the River-MImAS [9]. The comparison of results obtained from different assessment methods is convoluted, this highlighting the need for an unbiased and standardised protocol for hydromorphological characterisation.

The existing approaches are commonly implemented via *in*-*situ* mapping [8] or aerial imagery assessment [10]. The former relies on the expertise of the surveyor identifying hydromorphological features and does not allow for the objective re-assessment of records after survey completion. Moreover, due to practical time and cost constraint these surveys are difficult to repeat at high frequencies and are limited to accessible reaches [11,12,13,14]. Therefore, such assessments lack of spatial detail and do not capture the spatio-temporal variability within river reaches. In contrast, approaches based on aerial imagery rely on the visual or automated identification of key river characteristics from off-the-shelf imagery of generally 12.5 cm or 25 cm resolution. Here, the quality of the assessment depends upon the accuracy of the classification approach and the characteristics of the imagery, such as resolution and wavelength bands.

There are three types of image classification techniques [15]: object-based image analysis, unsupervised and supervised image classification. Object-based techniques rely on multi-resolution segmentation and are able to simultaneously generate objects of different shapes and scales by grouping pixels of similar characteristics. Unsupervised classification groups pixels based on their reflectance properties whereas supervised classification is based in the concept of segmenting the spectral domain into areas that can be associated with features of interest. The later method requires a training process by which representative samples of features of interest are identified and used to classify the entire image. There is a large array of algorithms for the task [15] such as maximum likelihood, Gaussian mixture models, minimum distance and networks of classifiers. Amongst all the existing supervised classification approaches, methods based on Artificial Neural Networks (ANNs) have been shown to enable image pattern recognition at particularly high precision with both coarse [16,17] and fine resolution imagery [18,19]. For example, [20,21,22] used ANNs to identify green canopy cover from background soil and shadows. ANNs have also been used successfully to map water bodies [23] and flood extent [24]. However, to the authors’ knowledge the usefulness of ANNs in the classification of river features as part of hydromorphological assessment has not been tested yet.

The use of image classification techniques for river mapping is well documented and has been applied successfully on hyperspectral imagery for the identification of hydraulic and habitat patterns [25], woody debris [14], channel substrate [26] and riparian vegetation [26]. Although hyper and multispectral bands are the preferred wavelength bands to classify hydromorphological features, they require exhaustive data processing algorithms and post-interpretation [26]. Spaceborne hyperspectral imagery (30 to 50 m ground resolution) does not offer the required resolution for detail river feature identification and fails to provide global spatio-temporal coverage. It may therefore be difficult to obtain off-the-shelf data for a given location and event (time) of interest. Airborne hyperspectral imagery can address this limitation but data capture can be expensive as it requires hired hyperspectral flights tailored to the objectives of the research. The spatial (ground) resolution of airborne hyperspectral imagery (typically above meter resolution) is significantly larger than typical high-resolution RGB photographs as a result of a lower number of photons per channel than imaging spectrometers [26]. Hyperspectral imagery may also be highly sensitive to imaging geometry (e.g., differences from the middle to the edge of the flight path) and environmental conditions (e.g., water vapour). In addition, hyperspectral imagery does not represent a snapshot of the area as pixel’s data is collected consecutively—*i.e.*, there is a lag between acquisitions of consecutive pixels. For airborne imagery, this translates into unequal pixel geometries and major issues in image rectification [26].

The classification of key features from RGB imagery has already been proved to be an efficient method for the automated identification of macrophytes [27]. This also holds true for vision based classification techniques for both geomorphic and aquatic habitat features [28] and is consistent with the excellent results shown for the characterisation of fluvial environments [29,30]. Recent studies have attempted to improve the results of existing classification methods by using Unmanned Aerial Vehicle (UAV) high resolution RGB aerial imagery. For example, [31] used UAV and ultra-light aerial vehicles imagery of resolutions between 3.2 cm and 11.8 cm to assess rates of vegetation recruitment and survival on braided channels in the Alps and [32] used UAV aerial imagery with resolutions from 1 cm to 10 cm to quantify the temporal dynamics of wood in large rivers.

The combination of UAV high resolution imagery and automated classification techniques for the identification of features within river environments has been documented by several authors [33,34]. In [33] supervised machine learning approaches were used to identify different types of macrophytes whereas in [34] standing dead wood presence in Mediterranean forests was mapped combining 3.2 to 21.8 cm resolution imagery and object oriented classification approaches.

The increased availability of low cost, vertical take-off UAV platforms and the increased legal requirement for improved river monitoring protocols make particularly attractive the used of RGB UAV high resolution aerial imagery for the development of a plausible, transferable and standardised framework for hydromorphological assessment. This solution could offer both timely (on-demand) and detailed (higher resolution) information than remote sensing imagery. Here, we extend the combined use of UAV high resolution RGB aerial imagery and ANNs to automatically classify all existing hydromorphological features along a 1.4 km river reach. The aim is to develop and assess a framework combining UAVs, high resolution imagery and ANNs for the unbiased characterisation of river hydromorphology. This is achieved through the following three core objectives:

i. To adapt existing ANN classification software for the identification of hydromorphological features,
ii. To assess the suitability of UAV high resolution aerial imagery for (i),
iii. To quantify the accuracy of the operational framework derived from (i) and (ii).

## 2. Experimental Section

### 2.1. Study Site

**Figure 1:**
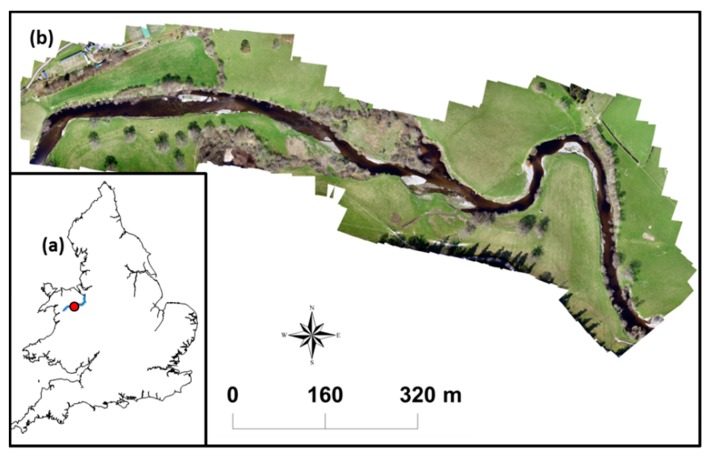
(**a**) Location of the study site along the river Dee near Bala, Wales, UK; (**b**) Detailed view of the study area.

The case study area is a 1.4 km reach in the upper catchment of the river Dee near Bala dam, Wales, UK (Figure 1a). The river Dee flows North-East from its origin in Dduallt (Snowdonia) into Bala lake to descend East to Chester and discharge in an estuary between Wales and the Wirral Peninsula in England. It defines the boundary between Wales and England for several miles from Bangor-on-Dee to Aldford. The catchment area of the 110 km long river covers 1816 km^2^, with the study site located approximately at 30 km from its origin. The fieldwork took place from the 20th to the 25th of April 2015 under low flow conditions and with a constant volumetric flow rate of 4.8 m^3^·s^−1^. The UAV imagery was collected on the 21 April 2015.

### 2.2. Sampling Design

**Figure 2:**
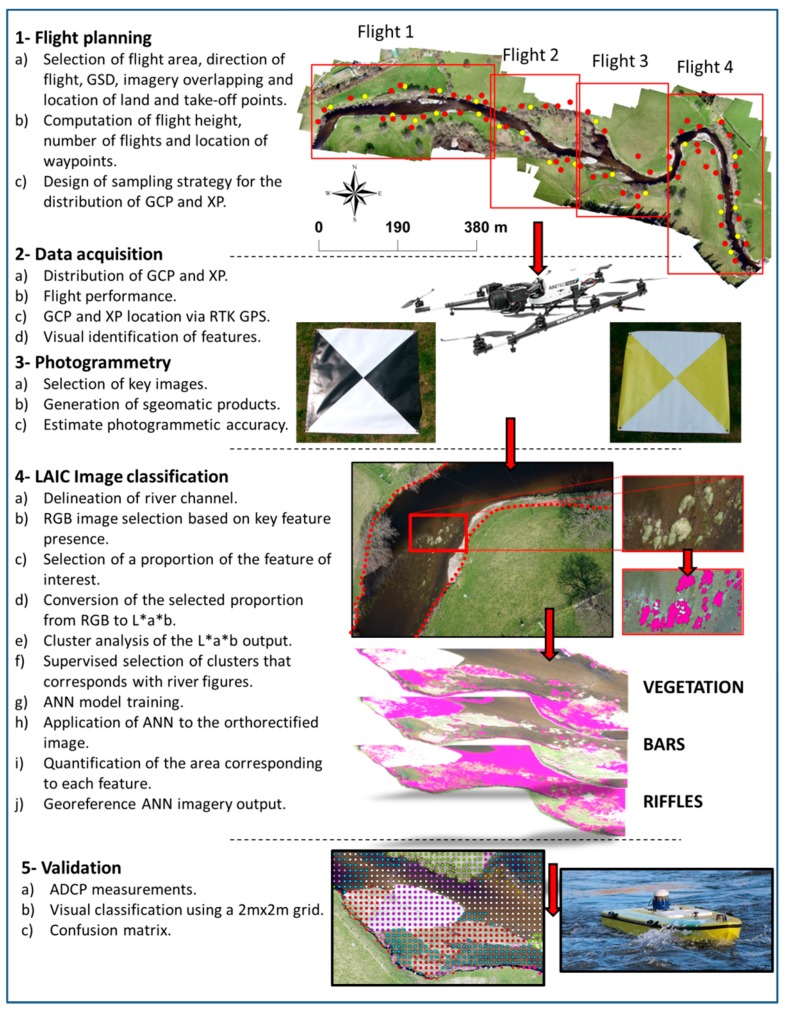
Workflow summarising the steps followed in the photogrammetry using Photoscan Pro and the image classification using the Leaf Area Index Calculation (LAIC) software, based on the workflows presented by [21,37], respectively. GDS, GCP and XP stand for Ground Sampling Distance, Ground Control Point (red points) and Check Point (yellow points), respectively.

**Figure 3:**
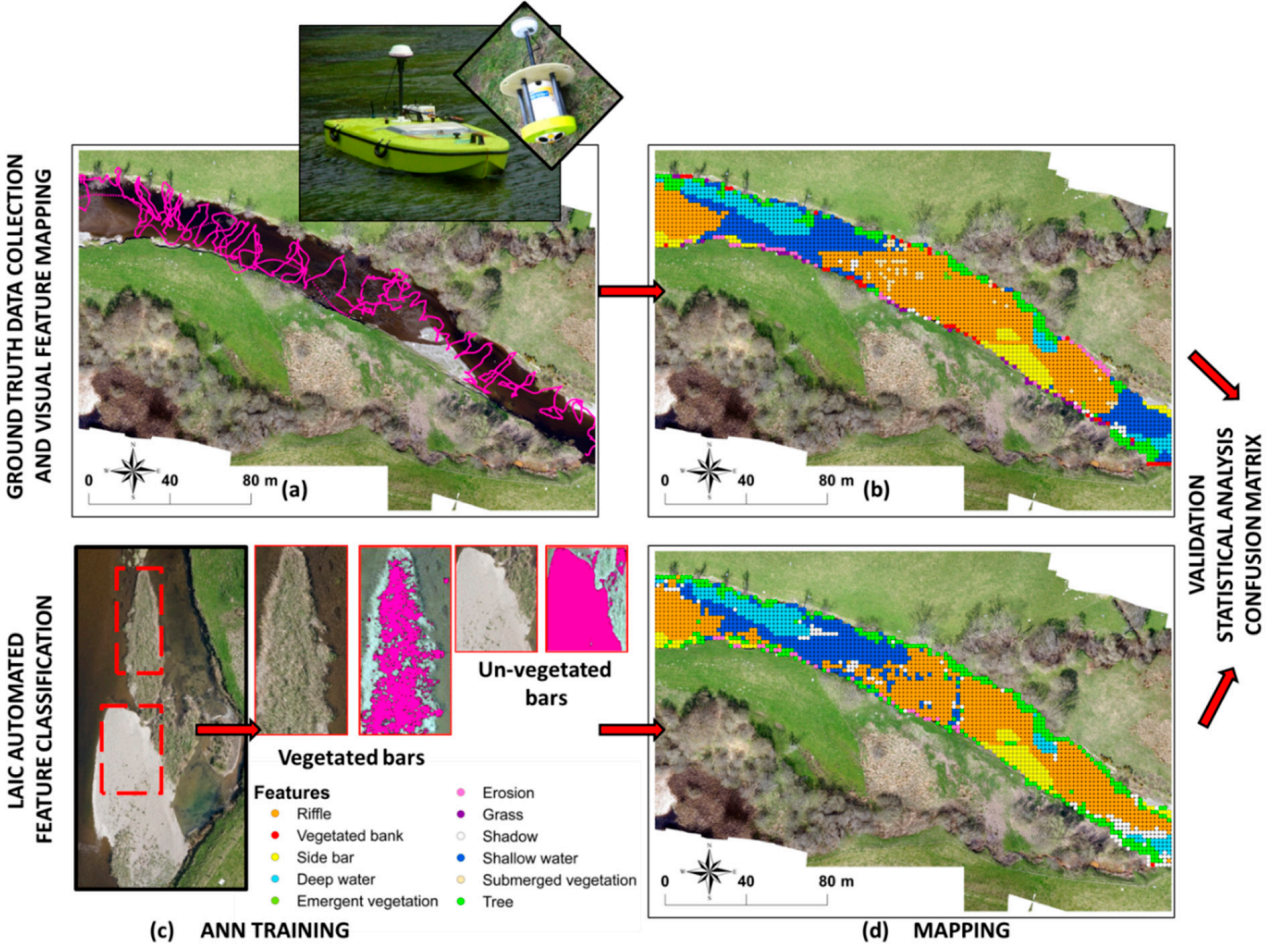
Detailed diagram of the workflow for the Leaf Area Index Calculation (LAIC) image classification and validation based on [18] (**a**–**d**). (**a**) 300 m section within the reach showing the ADCP measurements obtained along with a detailed image of the radio control boat and ADCP sensor used; (**b**) Map showing the hydromorphological features obtained from visual identification on a 2 m × 2 m regular grid; (**c**) Examples of sections selected for and outputs obtained from the Artificial Neural Network (ANN) training; (**d**) Map showing the hydromorphological feature classification obtained with ANN on a 2 m × 2 m regular grid.

A total of 60 1 m × 1 m Ground Control Points (GCPs) were distributed uniformly within the flying area (Figure 2) to obtain parameters for external orientation [28,35]. The centroid of each 1 m × 1 m white 440 g PVC GCP was established via its square diagonals. Opposite facing triangles were painted in black to facilitate centroid identification (Figure 2). GCPs were pinned with pegs to the ground through four metallic eyelets. The locations of the GCP centroids were obtained from a Leica GS14 Base and Rover Real Time Kinematic (RTK) GPS with a positioning accuracy of 1–2 cm in the X, Y and Z dimensions. Further 25 yellow and white check points (XPs, Figure 2) were set to quantify image coregistration model errors [35] using the same deployment strategy as for the GCPs. Velocity and depth measurements within the channel were obtained using a SonTek RiverSurveyor M9 Acoustic Doppler Current Profiler (ADCP) mounted on an ArcBoat radio control platform [36] (Figure 3). The reach was sampled following a bank to bank zig-zag pattern to capture the spatial variability in channel depth and water velocity.

### 2.3. UAV and Sensor

Aerial imagery in the visible spectrum was acquired via an AsTec Falcon 8 octocopter (ASCTEC, Krailling, Germany) equipped with a Sony Alpha 6000 camera (Sony Europe Limited, Weybridge, Surrey, UK) and a u-blox LEA 6S GPS. The 0.77 m × 0.82 m × 0.12 m octocopter has a vertical take-off weight of 1.9 kg, where the sensor payload accounts for 0.34 kg. Fully charged Lithium Polymer (LiPo) batteries (6250 mAh) provided a maximum flight time of 22 min. The Falcon 8 can tolerate wind speeds of up to 15 m·s^−1^—a threshold that was never exceed during data collection. The weather conditions during the flight, based on Shawbury meteorological aerodrome report (METAR), presented surface winds of speeds between 1 m·s^−1^ and 3 m·s^−1^ and directions varying from 60° to 350°, with prevailing visibility up to 10,000 m AMSL and a few clouds at 12,800 m AMSL.

The study area was surveyed through four consecutive flight missions (Figure 2). Full spatial coverage was ensured through the combination of longitudinal and cross-sectional multipasses. The flight was pre-programmed with the AscTec Navigator software version 2.2.0 for a number of waypoints to achieve 60% along track and 80% across track image overlap for the camera parameters described in Table 1 and a flight height of 100 m. The resulting ground sample distance (GSD) was 2.5 cm. Each waypoint represented the centre of a frame and had associated GPS coordinates as well as yaw, pitch and roll information. The UAV was held stationary at waypoints and transited between them at a speed of 3 m·s^−1^. The operator was a fully qualified RPQ-s (Small UAV Remote Pilot Qualification) pilot and followed Civil Aviation Authority (CAA) legislation CAP393 [38] and CAP722 [39].

The Sony Alpha 6000 camera (Table 1) relies on complementary metal oxide semiconductor (CMOS) image sensor technology. The APS-C 2.82 cm (1.11 inches) diameter CMOS sensor provides images of 24.3 effective megapixels (6000 × 4000 pixels). The colour filter type used was RGB.

**Table 1:** Key characteristics for the Sony Alpha 6000 complementary metal oxide semiconductor (CMOS) sensor.

Characteristics	Sony Alpha 6000
Sensor (Type)	APS-C CMOS Sensor
Million Effective Pixels	24.3
Pixel Size	0.00391 mm
Image size (Columns and Rows)	6000 × 4000
Lens	24–75 mm (35 mm)
Focal	3.5–5.5
ISO range	100–51,200

### 2.4. Photogrammetry

A total of 394 frames out of 746 were selected for the photogrammetric analysis. The selection was based upon image quality and consecutive spatial coverage. Photoscan Pro version 1.1.6 (Agisoft LLC, St. Petersburg, Russia) was used to generate an orthoimage. Figure 2 summarises the workflow adapted from [37]. The coordinates for each of the GCPs were used to georeference (scale, translate and rotate) the UAV imagery into the coordinate system defined by the World Geodetic System (WGS84) and minimise geometric distortions. Image coregistration errors were estimated at each GCP as the difference between the positions measured through RTK GPS and the coordinates derived from the imagery. A combined measure of error for *x* and *y* is obtained from Equation (1):

(1) $$\text{RMSE}=\sqrt{\frac{\sum_{j=1}^{N}[{\left({\hat{x}}_{j}-x_{j}\right)}^{2}+{\left({\hat{y}}_{j}-y_{j}\right)}^{2}]}{N}}$$

where RMSE is the Root Mean Squared Error, $\hat{x}$ and $\hat{y}$ are the image derived coordinates at location *j*, x and y are the associated RTK GPS positions and *N* is the number of points assessed. If the XPs are assessed independently, their RMSE becomes an unbiased validation statistic.

The overall process to obtain the geomatic products (*i.e.*, point cloud, orthoimage and digital terrain model) required 12 h of processing time based on the performance of a computer with an Intel Core i7-5820K 3.30 GHz processor, 32 Gb RAM and 2 graphic cards (NVIDIA Geoforce GTX 980 and NVIDIA Qadro K2200).

### 2.5. Image Classification

The hydromorphological feature classification was implemented with the Leaf Area Index Calculation (LAIC) software (Figure 2 and Figure 3). LAIC is a MATLAB-based supervised ANN interface designed to discriminate green canopy cover from ground, stones and shadow background using high resolution UAV aerial imagery [20,21]. In brief, LAIC relies on clustering techniques to group the pixels from high resolution aerial imagery based on CIELAB characteristics. From the three parameters describing the CIELAB space [40,41] (*i.e.*, lightness (*L*), green to red scale (*a*) and blue to yellow scale (*b*)) only *a* and *b* are taken into account by the clustering algorithm. For this purpose, RGB collected at representative waypoints was transformed to L* a* b* colour-space. A k-means clustering algorithm of the RGB levels on the L* a* b* colour-space transformed imagery was then implemented. The analysis groups pixels into *k*-clusters (*k*) with similar red/green (*a*) and yellow/blue (*b*) values. The number of clusters (*k*) depends on the feature being identified and was determined following an iterative process that increased *k* by one up to a maximum of ten clusters until visually satisfactory results were obtained. Within this context, visually satisfactory results required the image outputs (Figure 3) to show that the feature of interest (Table 2) had been adequately identified. This supervised method was used as a basis to calibrate a Multilayer Perceptron (MP) ANN, which was applied to the remaining images. The calibration process is highly-time consuming and therefore, only a small section of the imagery can be used for this purpose. Key to the success of the ANN was the adequate selection of the small proportion of imagery used for the ANN calibration and training process. These were chosen based on the presence of such features in at least 50% of the selected area. Here, we looked at the clarity of the colours as well as the contrast in *a* and *b*. Images with shadows or including features that could be confused with the one of interest were not selected.

The basic MP ANN is a simple, binary linear classifier that, once trained places patterns into one of the two available classes by checking on which side of the linear separating surface they lay [15]. In this study, we used a more complex form of MP ANN [16] based on three consecutive layers named input, hidden and output, hereafter. Each layer is composed of inter-connected nodes, also known as neurons. The results from the cluster analysis are input into the first layer which performs linear combinations of the input parameters to give a set of intermediate linear activation variables. In turn, these variables are transformed by non-linear activation functions in the hidden layer where a secondary layer of weights and biases provides a set of activation values. These are then fed into the output layer to obtain the final output values. The weights were adjusted via an iterative back propagation process based on the comparison of the outputs with target features characterised in the training process. The network is initialised with a set of weights. For each training pixel, the output of the network is estimated using the structure beforehand mentioned. The weights are then corrected based on the resulting outcome. This iteration process stops once an optimisation algorithm has been satisfied. In this study, a quasi-Newton optimisation algorithm aiming at minimising the estimation error [16] was used in the training process to ensure the non-linear structure of the MP ANN was accounted for [20].

The number of outputs nodes depends on how the outputs are used to represent the features. In this study, the number of output processing elements is the same as the number of training classes. Each class was trained separately and therefore, there was only one output node—with value one when the RGB values corresponded to the selected feature and zero for any other instances [20]. The software outcomes provided (i) a classified map of the study area and (ii) estimates of the areas allocated to each of the classified features. The ANN was used to recognise the key features described in Table 2. These features are used by Governmental agencies [8] and environmental scientists [35,37] alike to describe homogeneous areas of substrate, water and vegetation within the reach.

**Table 2:** Hydromorphological features identified within the study area based on [8].

Feature		Description
Substrate Features	Side Bars	Consolidated river bed material along the margins of a reach which is exposed at low flow.
	Erosion	Predominantly derived from eroding cliffs which are vertical or undercut banks, with a minimum height of 0.5 m and less than 50% vegetation cover.
Water Features	Riffle	Area within the river channel presenting shallow and fast-flowing water. Generally over gravel, pebble or cobble substrate with disturbed (rippled) water surface (*i.e.*, waves can be perceived on the water surface). The average depth is 0.5 m with an average total velocity of 0.7 m·s^−1^.
	Deep Water (Glides and Pools)	Deep glides are deep homogeneous areas within the channel with visible flow movement along the surface. Pools are localised deeper parts of the channel created by scouring. Both present fine substrate, non-turbulent and slow flow. The average depth and is 1.3 m and the average total velocity is 0.3 m·s^−1^.
	Shallow Water	Includes any slow flowing and non-turbulent areas. The average depth is 0.8 m with an average total velocity of 0.4 m·s^−1^.
Vegetation Features	Tree	Trees obscuring the aerial view of the river channel.
	Vegetated Side Bars	Side bar presenting plant cover in more than 50% of its surface area.
	Vegetated Bank	Banks not affected by erosion.
	Submerged Free Floating Vegetation	Plants rooted on the river bed with floating leaves.
	Emergent Free Floating Vegetation	Plants rooted on the river bed with floating leaves on the water surface.
	Grass	Present along the banks as a result of intense grazing regime.
Shadows		Includes shading of channel and overhanging vegetation.

To simplify the complexity of the ANN implementation, only the imagery within the area defined by the bank channel boundary (46,836 m^2^) was considered in the overall classification. The 2.5 cm orthoimage was divided into 20 tiles for processing via LAIC. This decreased the central processing unit (CPU) demands during feature recognition. The tiles were georeferenced and mosaicked together to obtain a complete classified image for the study area. The overall classification process with LAIC was undertaken in less than 7 h.

### 2.6. Radio Control Boat and ADCP Sensor

Water depths and velocities were obtained with an ADCP and used to define an informed threshold between deep and shallow waters (Table 2). Water depths were used in the validation process whereas water velocities were only used for descriptive purposes in Table 2. ADCPs are hydro-acoustic sensors that measure water velocities and depth by transmitting acoustic pulses of specific frequency [42]. ADCPs deployed from a moving vessel provide consecutive profiles of the vertical distribution of 3D water velocities and depth along the vessel trajectory. The ADCP used in this study was a RiverSurveyor M9 ADCP with a SonTek differentially corrected GPS [43]. The ADCP data were collected at a frequency of 1 Hz and an average boat speed of 0.35 m·s^−1^.

### 2.7. Validation

Hydromorphological features (Table 2) were mapped along the reach during a “walk-over” survey. A 2 m × 2 m grid was overlaid onto the othoimage in a Geographical Information System (GIS) environment (ArcGIS 10.3, Redlands, CA, USA). The hydromorphological feature classes were visually assigned to each of the 13,085 points defined by the regular grid. The visual classification was aided by 118 documented colour photographs and 470 RTK GPS measurements providing the exact locations of the hydromorphological features described in Table 2. The visual point class outputs were compared to those obtained from the ANN classified image via a confusion matrix [44], where the visual classification was considered to be the ground truth. Measures of accuracy (AC), true positive ratios (TPR), true negative ratios (TNR), false negative ratios (FNR) and false positive ratios (FPR) were derived for each hydromorphological feature (*i*) as follows:

(2) $$\text{AC}=\frac{\text{TN}+\text{TP}}{\text{TN}+\text{TP}+\text{FN}+\text{FP}}$$

(3) $${\text{TPR}}_{i}=\frac{{\text{TP}}_{i}}{{\text{FN}}_{i}+{\text{TP}}_{i}}$$

(4) $${\text{TNR}}_{i}=\frac{{\text{TN}}_{i}}{{\text{TN}}_{i}+FP_{i}}$$

(5) $${\text{FNR}}_{i}=\frac{{\text{FN}}_{i}}{{\text{FN}}_{i}+{\text{TP}}_{i}}$$

(6) $${\text{FPR}}_{i}=\frac{{\text{FP}}_{i}}{{\text{TN}}_{i}+{\text{FP}}_{i}}$$

where TP (true positives) is the number of points correctly identified as class *i*, FN (false negatives) is the number of points incorrectly rejected as class *i*, TN (true negatives) is the number of points correctly rejected as class *i* and FP (false positives) is the number of points incorrectly identified as class *i*.

TPR, TNR, FNR and FPR are estimated for each of the features of interest whereas AC is a single value of overall classification performance. AC as well as all the ratios beforehand mentioned range from 0 to 1 or 0% to 100% when reported in percentages. Both true positives (TP) and true negatives (TN) estimate the number of points that have been correctly identified or rejected to fall within a particular class. Therefore, TPR and TNR quantify the power of LAIC at classifying features correctly when compared to the ground truth. Both false negatives (FN) and false positives (FP) estimate the number of points that have been falsely rejected or falsely identified to fall within a particular class. Hence, FNR and FPR show the rates of misclassification when compared to the ground truth values.

## 3. Results

The image coregistration model errors estimated from the GCP and XP positions were consistent and within the proposed thresholds reported by [35]. The average error was below 1.7 cm (X = 1.1 cm, Y = 1.0 cm and Z = 1.6 cm) for the GCP and below 2 cm (X = 0.4 cm, Y = 2.0 cm and Z = 0.53 cm) for XP. The RMSEs were 1.5 cm and 0.09 cm for GCP and XP, respectively. The accuracy of the ANN classification (Equation (2)) was 81%, meaning that a total of 10,662 points out of 13,085 were classified correctly, with the majority of classes showing a TPR above 85% (Table 3 and Table 4). The ANN reached a solution for the backpropagation process under 60 iterations.

**Table 3:** Confusion matrix of visual classification (VC) *versus* Artificial Neural Network (ANN) classification. Feature codes have been abbreviated as follows: side bars (SB), erosion (ER), riffle (RI), deep water (DW), shallow water (SW), tree (TR), shadow (SH), vegetation (VG), vegetated bar (VB), vegetated bank (VK), submerged vegetation (SV), emergent vegetation (EV) and grass (GR). GE stands for georeferencing error.

ANN Classification											
Feature	VC	SB	ER	RI	DW	SW	TE	SH	VG	GE	Total
SB	1334	1097	-	8	-	2	-	10	214	3	1334
ER	287	-	22	13	1	3	-	10	238	-	287
RI	3339	-	1	2717	-	318	-	219	76	8	3339
DW	2082	-	-	60	1927	54	-	8	29	4	2082
SW	2573	-	-	262	80	1514	-	493	217	7	2573
TR	1755	-	-	76	1	29	496	135	1013	5	1755
VB	299	-	-	-	-	-	-	-	299	-	299
VK	313	-	10	-	6	-	-	15	281	1	313
SV	468	-	-	160	-	125	-	46	135	2	468
EV	71	-	1	9	-	2	-	1	58	-	71
GR	344	-	-	-	-	-	-	-	343	1	344
SH	220	-	4	-	-	-	-	180	31	-	220
Total	13,085	1097	38	3305	2015	2052	496	1117	2934	31	13,085

**Figure 4:**
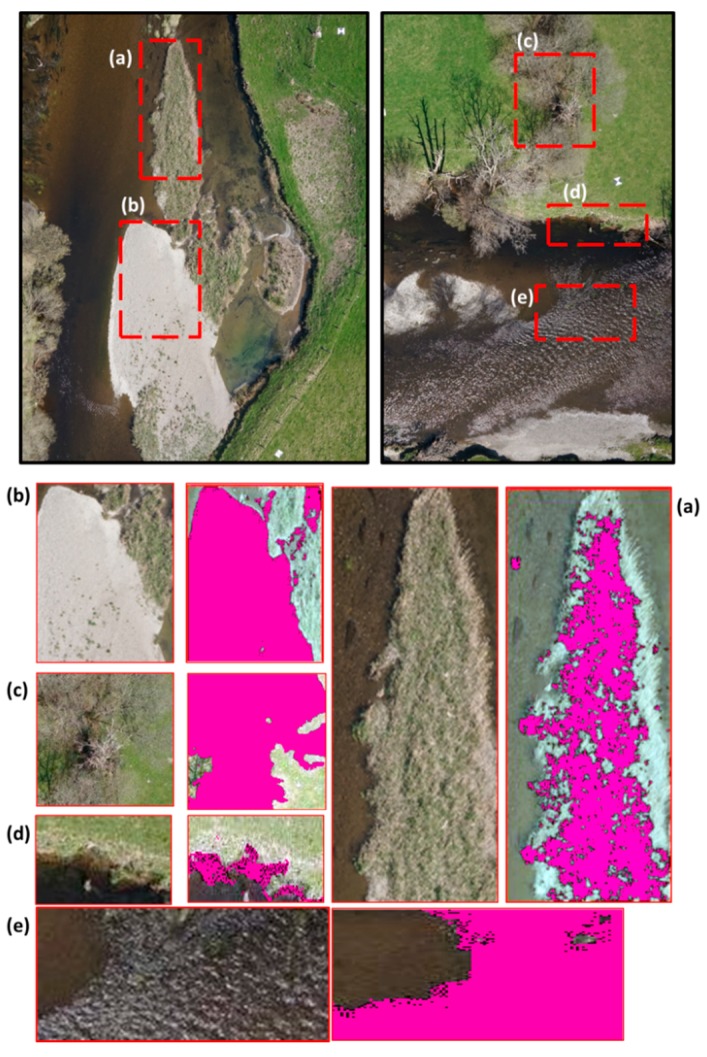
Example of trained outputs for (**a**) Vegetation in bars; (**b**) Side bars with no vegetation; (**c**) Trees; (**d**) Erosion and (**e**) Riffle. The outputs portray the portion of the imagery selected for analysis and the pixels selected (pink) by the cluster algorithm.

The georeferencing errors derived from tile misalignment of LAIC outputs accounted for 0.2% of the totality of points. The TPR of shadow identification was above 80% with no significant misclassification results. FNR and FPR were below 20% in all cases except for erosion, shallow water and submerged vegetation feature classes. These values are very low and can be explained by misclassification errors described in the following sections. The overall study area (46,836 m^2^) was dominated by a combination of riffle (31%) and deep water (24%) features (Table 5). Figure 4 presents an example classification output for some of the features identified within a selected tile.

**Table 4:** True positive ratio (TPR), true negative ratio (TNR), false negative ratio (FNR) and false positive ratio (FPR) for each of the class features identified by the Artificial Neural Network (ANN) within the river reach.

Feature Identification (ANN)		TPR	TNR	FNR	FPR
Substrate Features	Bars	0.822	0.765	0.178	0.000
	Erosion	0.077	0.786	0.923	0.001
Water Features	Riffle	0.814	0.756	0.074	0.060
	Deep Water	0.926	0.741	0.074	0.008
	Shallow Water	0.588	0.815	0.412	0.051
Vegetation	Trees	0.860	0.757	0.140	0.082
	Vegetated Bar	1.000	0.765	0.000	0.082
	Vegetated Bank	0.898	0.767	0.102	0.082
	Submerged Vegetation	0.288	0.788	0.712	0.082
	Emergent Vegetation	0.817	0.770	0.183	0.082
	Grass	0.997	0.750	0.003	0.082
Shadow		0.818	0.770	0.182	0.073

**Table 5:** Areas for each of the features estimated from the Artificial Neural Network (ANN) classification.

Feature	Area (m^2^) ANN
Bars	4992
Erosion	338
Riffle	12,758
Deep Water	10,008
Shallow Water	7977
Vegetation	10,080
Shadow	683
Total	46,836

### 3.1. Substrate Features

**Figure 5:**
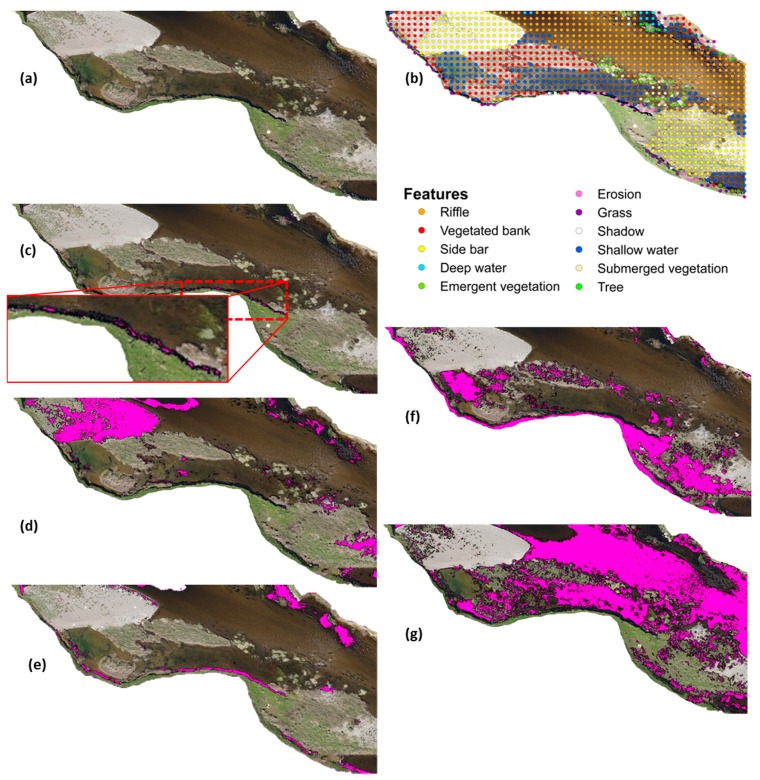
Example of Artificial Neural Network (ANN) classification outputs obtained with the Leaf Area Index Calculation (LAIC) for a selected portion of the orthoimage. Pixels elected within each class are shown in pink. (**a**) Original image; (**b**) Visual classification for the points defined by a 2 m × 2 m regular grid; (**c**) Erosion; (**d**) Side bars; (**e**) Deep water; (**f**) Vegetation (all classes); (**g**) Riffles. The image is not to scale.

**Figure 6:**
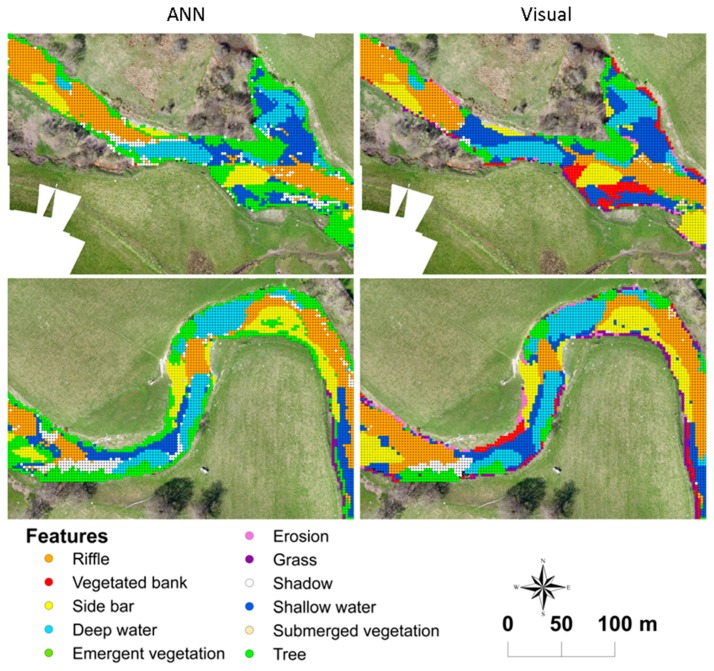
Classification outputs at each of the points defined by a 2 m × 2 m regular grid obtained with (**Left**) The Leaf Area Index Calculation (LAIC) Artificial Neural Network (ANN) and (**Right**) The visual identification for two sections within the study reach.

Erosion features (Figure 4d) had the lowest TPRs, scoring only 8% (Table 3). Many of the eroded banks within the reach were vertical cliffs and not visible from a 2D planar aerial view (Figure 5c). The majority of these areas presented grass up to the edge of the cliff and vegetated sediment deposits at the bottom. The ANN classification therefore defaulted to vegetation or shadow (generated by the cliff) in the majority of observations (Table 3 and Figure 6).

Side bars (Figure 4b and Figure 5d) had a TPR and TNR above 76% (Table 3). Misclassification occurred when vegetation appeared within the bar—the class defaulting to vegetation in all instances (Figure 5f and Figure 6). The ANN was able to correctly identify the vegetation (*i.e.*, TPR for vegetated bar was 100% in Table 3) but unable to specify the feature where the vegetation was encountered (e.g., bank, bar or tree spring shot). If these FN were considered TP, the TPR ratio for side bars increased to 98%.

### 3.2. Water Features

The ANN based classification achieved a TPR and TNR above 75% when identifying riffles. However, for LAIC to correctly classify a point as a riffle (Figure 4e), this feature had to coincide with shallow water and a rippled water surface (Figure 6). Confusion of riffles for shallow water occurred when rippled surfaces were not present within the riffle. All the points thus misclassified fell within close proximity of riffles and could be considered as TP, increasing TPR to 91%. Misclassification as shadow occurred in (i) areas where mossy submerged vegetation presented a deep brown colour and could not be distinguished from the channel bed or (ii) shallow water areas presenting a darker colour due to sedimentation.

Deep water (Figure 5e) showed the highest TPR at 92% with the majority of misclassified features falling under riffles and occurring along the transition from riffles to pools. Similarly, confusion with shallow waters occurred in the transition zone from deep to shallow areas (Figure 5e and Figure 6). Deep water was classified as vegetation near the banks. This was primarily due to georeferencing errors when mosaicking LAIC tail outputs.

Shallow waters presented the same errors as those already described beforehand. Here, shallow water with rippled water surface was automatically classified as riffles. This could not be identified as an error in classification but as higher resolution in feature detection than expected. When correcting for this effect, the TPR increased to 69% for shallow water, with a rate of TN equal to 81%. Misclassification of shallow waters as shadows primarily occurred in areas below trees or water areas obscured by brown submerged vegetation. In the first instance, we assumed that the visual classification was inaccurate whereas in the second case vegetation generated dark undistinguishable and even shadow patterns.

### 3.3. Vegetation

All vegetation classes combined resulted in a TPR of 81% with all the individual classes above 80% except for submerged vegetation (29%). Submerged vegetation was generally classified as either shallow water or riffle whereas classification as shadow primarily appeared in areas where the vegetation presented a brown colour due to sediment deposits.

Vegetated banks were primarily confused with erosion or shadows. Erosion corresponded to sediment deposits where senescent vegetation was present whereas shadows were the result of LAIC detecting features at a higher level of resolution than that obtained from visual classification. In all instances LAIC correctly identified the shadows generated by the vegetation. For the tree feature class, all misclassifications as riffles or shallow water accounted for LAIC extracting the riffle class from the water that was visible between branches. In general, trees misclassified as shadows corresponded to shadows generated by the tree branches. No significant misclassification errors were identified for emergent free floating vegetation, grass and vegetation in bars, with TRP values above 99%.

## 4. Discussion

This paper presented an operational UAV based framework for the identification of hydromorphological features using ANNs and high resolution RGB aerial imagery. To the authors’ knowledge, this is the first study looking at the development of a framework for the automated classification of all hydromorphological features within a river reach. The framework proved to be accurate at 81% and enabled the identification of hydromorphological features at a higher level of detail than that provided by the ground truth data. For example, it allowed the separation of trees from the grass or water underneath, as well as the recognition of hydraulic units (e.g., rippled water surface).

The approach is: (i) transferable, because once the ANN has been trained, it can be applied to other river sections without direct supervision; (ii) unbiased, because it enables the objective classification of hydromorphological features and (iii) flexible, because it is able to identify the multiple hydromorphological features that can be attributed to a single point. Hydromorphological features are not discrete units but an interaction of multiple layers with fuzzy limits that reflect the spatial complexity of river environments [45]. Points can therefore fall simultaneously within multiple feature classes (e.g., riffles and submerged vegetation). LAIC has the potential to identify multiple classes for a single point based on the hydraulic, habitat or vegetation characteristics observed through the selection of different number of clusters during the ANN training.

Shadows within the imagery pose one of the primary barriers to correct feature identification. This issue has been recognised in previous work by the authors [21,22]. The overall ANN accuracy (81%) could therefore be improved through detailed flight planning that aims at minimising the presence of shadows. Thoughtful selection of the time of the flight to avoid shadows will also increase the potential for erosion identification. Flight optimisation also needs to consider (i) seasonality and (ii) flight direction. Winter flights present the advantage of exposing the totality of the river channel whereas spring and summer flights will not enable the identification of in-channel features under dense vegetation cover. However, fully developed vegetation will expose different green RGB totalities and allow LAIC to identify plant species. The optimisation of the flight direction is essential for wide area mapping (e.g., at sub-catchment scale). This will reduce the flight time, ensure all the imagery is collected under similar light conditions, minimise the number of frames and the CPU load required to build the orthoimage. Previous research with UAVs in rivers [46] have proved longitudinal multipasses to be more efficient than cross-sectional ones without compromising the quality of the photogrammetric process.

The overall approach here presented is based on near real-time RGB imagery of higher resolution than multi- and hyperspectral imagery from manned aircraft used in the past for similar purposes [14,25]. It complements existing tools for characterising rivers or fluvial features [47] such as the Fluvial Information System [48] or the “FluvialCorridor” [49]. Similarly, it provides the basis for the comparison and harmonisation of results obtained from the exhaustive list of available hydromorphological assessment methodologies [50]—a much sought outcome required by the WFD for intercalibration purposes [51]. Although time consuming, the *k*-means based ANN approach was preferred to visual photointerpretation as it provides an objective way for the classification of river environments that could be automatically applied to the national river network. Some of the time consuming steps only need to be carried out occasionally. For example, the ANN training is a one-off process that only needs to be repeated whenever significant changes in environmental conditions occur (e.g., river turbidity or weather pattern variation). Other time-consuming steps such as frame selection or GCP location can be optimised thus significantly reducing the time demand.

The technique relies on a minimum threshold of illumination and limited presence of shadows. The ideal flying time for data capture purposes is therefore solar noon. In this study, all the frames were captured on the same day under stable weather conditions and within a 6 h interval. However, these minimal changes in sun position and orientation could result in an increased number of miss-classified features along the downstream sections of the reach due to an increased presence of shadows. Illumination is not a key factor affecting the classification, as long as a minimum level of brightness is present. This is because LAIC bases the clustering technique only on the a*b parameters of the CIELAB space [40,41], without taking into account the luminosity (L).

It is important to note that for the adoption of the framework at national and international level, several operational limitations for small UAVs should be addressed. This includes battery life endurance, platform stability as well as standardised and flexible international airspace regulatory frameworks. The ANN approach already provides a fast processing platform for the recognition of patterns from UAV high resolution aerial imagery, with the UAV platforms presenting the main limitations for large scale mapping. Octocopters such as the one used in this study have the ability to hover over target areas to provide high resolution still imagery. Key to the UAV high performance is the design-integration of a gimbal that effectively negate pitch, yaw and roll. This camera gimbal allows the capture of nadir images to perform a highly-accurate photogrammetric workflow. Fix wing platforms can be a better alternative to cover larger areas but this may come at a cost to imagery quality and resolution. Further considerations to increase the accuracy of the ANN based feature identification relates to the reduction of georeferencing and misalignment mosaic errors (2%) from LAIC outputs. This issue can be addressed by ensuring the outputs generated are automatically georeferenced and ready to upload into a GIS platform.

## 5. Conclusions

The ANN based framework herein described for the recognition of hydromorphological river features relies heavily on the use of UAVs for the collection of high resolution RGB true colour aerial photography. The approach presented provides robust automated classification outputs of river hydromorphological features. The Artificial Neural Network (ANN) Leaf Area Index Calculation (LAIC) software used for pattern recognition enabled identification of hydromorphological features at a higher level of detail than that derived from visual observation. The framework leverages the use of UAVs for environmental policy implementation and demonstrates the potential of ANNs and RGB true colour imagery for precision river monitoring and management. Key advantages for its large-scale implementation rely on its flexibility, transferability and unbiased results. Time-consuming tasks within the framework can be optimised to reduce CPU demand and processing time. Further work should look at enhancing the operational thresholds of small UAVs by for example, increasing battery live or increasing overall stability under gusty conditions.
